# The *Oncojanus* Paradigm of Respiratory Complex I

**DOI:** 10.3390/genes9050243

**Published:** 2018-05-07

**Authors:** Giulia Leone, Houda Abla, Giuseppe Gasparre, Anna Maria Porcelli, Luisa Iommarini

**Affiliations:** 1 Dipartimento di Farmacia e Biotecnologie (FaBit), Univeristà di Bologna, 40126 Bologna, Italy; giulia.leone5@studio.unibo.it (G.L.); houda.abla2@unibo.it (H.A.); iommarini.luisa@gmail.com (L.I.); 2 Dipartimento di Scienze Mediche e Chirurgiche (DIMEC), Università di Bologna, 40138 Bologna, Italy; giuseppe.gasparre@gmail.com; 3 Centro Interdipartimentale di Ricerca Industriale (CIRI) Scienze della Vita e Tecnologie per la Salute, Università di Bologna, 40100 Bologna, Italy

**Keywords:** respiratory complex I, mtDNA, mitochondria, mtDNA mutations, cancer, tumor progression, *oncojanus*

## Abstract

Mitochondrial respiratory function is now recognized as a pivotal player in all the aspects of cancer biology, from tumorigenesis to aggressiveness and chemotherapy resistance. Among the enzymes that compose the respiratory chain, by contributing to energy production, redox equilibrium and oxidative stress, complex I assumes a central role. Complex I defects may arise from mutations in mitochondrial or nuclear DNA, in both structural genes or assembly factors, from alteration of the expression levels of its subunits, or from drug exposure. Since cancer cells have a high-energy demand and require macromolecules for proliferation, it is not surprising that severe complex I defects, caused either by mutations or treatment with specific inhibitors, prevent tumor progression, while contributing to resistance to certain chemotherapeutic agents. On the other hand, enhanced oxidative stress due to mild complex I dysfunction drives an opposite phenotype, as it stimulates cancer cell proliferation and invasiveness. We here review the current knowledge on the contribution of respiratory complex I to cancer biology, highlighting the double-edged role of this metabolic enzyme in tumor progression, metastasis formation, and response to chemotherapy.

## 1. Introduction

**Figure 1:**
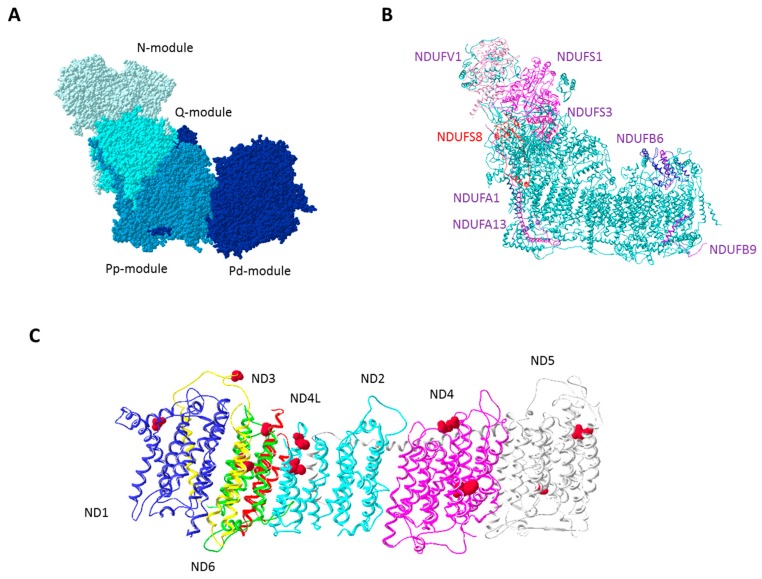
Complex I subunits involved in tumor biology. (**A**) Mammalian respiratory complex I (CI) structure based on the structure reported by Fiedorczuk et al. [15], (PDB ID: 5LNK). Different functional modules (NADH (N), quinone (Q) and proton-pumping (P) modules) are shown in shades of blue. The P-module is divided in Pp (proximal P module) and Pd (distal P module) following the most recent nomenclature [2]. (**B**) The CI nuclear subunits that have been found differentially expressed in tumors compared to normal tissues or cells. Downregulated subunits are highlighted in shades of purple, while NDUFS8, the only subunit found upregulated is colored red. (**C**) Mitochondrial DNA encoded subunits (ND1-6 and ND4L) of the membrane arm. Amino acid substitutions induced by missense mutations reported in the text and involved in tumor biology are shown as spheres and are colored red.

The mitochondrial oxidative phosphorylation (OXPHOS) system is the major site of energy production in eukaryotic cells and is composed of four respiratory complexes (complex I, II, III, and IV) and F_o_F_1_–adenosine triphosphate (ATP) synthase organized in functional supramolecular structures, such as dimers of single complexes or supercomplexes formed by the association of different complexes [1]. Among these enzymatic giants, respiratory complex I (CI) (also referred to as nicotinamide adenine dinucleotide (NADH): ubiquinone oxidoreductase/EC.1.6.5.3) is the largest, being composed of 44 subunits, seven of which (ND1–6 and ND4L) are encoded by mitochondrial DNA (mtDNA). The remaining 37 subunits are encoded by the nuclear DNA (nDNA), synthetized in the cytosol, and imported into the mitochondria where they associate with mtDNA-encoded subunits, form subcomplexes, and then assembly into the functional enzyme [2]. This process is mediated by several chaperones required for correct assembly but are dissociated from the mature complex and do not affect its function [3]. From a structural point of view, CI shows a slightly opened L-shape structure, in which two arms can be identified: the membrane embedded hydrophobic arm composed by both mtDNA- and nDNA-encoded subunits and the hydrophilic arm, exclusively composed by nDNA-encoded subunits, which protrudes into the mitochondrial matrix [4] (Figure 1A). Three functional modules can be clearly identified in the holoenzyme: (i) the hydrophilic NADH (N)-module oxidizes NADH, (ii) the hydrophilic quinone (Q)-module provides electron transfer through the Fe–S clusters to ubiquinone, and (iii) the hydrophobic membrane-embedded proton pumping (P)-module undergoes conformational changes to perform proton pumping in the mitochondrial intermembrane space [5]. The main function of CI is to couple proton translocation with electron transfer from NADH to ubiquinone, contributing to generation of the mitochondrial membrane electrochemical gradient, the driving force for ATP synthesis by F_o_F_1_–ATP synthase. As a byproduct of the electron transfer process, CI generates reactive oxygen species (ROS) having two sites accessible to O_2_ where formation of superoxide anion may occur, namely the NADH and the ubiquinone binding sites [6].

Complex I enzymatic defects may arise from the occurrence of pathogenic mutations in CI structural genes or assembly factors [7], in genes coding for proteins involved in CI subunits import or maturation, and in all the factors involved in the molecular machinery necessary for mtDNA replication, transcription, or translation [8]. In addition, CI impairment can be also induced or worsened by exposure to toxicants or drugs [9,10,11]. By performing its function, respiratory CI actively controls the cellular energetic and redox balance, providing a crucial checkpoint of cell metabolism, proliferation, and survival. Hence, it is not surprising that respiratory CI dysfunctions have been involved in the pathogenesis of several human diseases, including mitochondrial disorders [12] and neurodegenerative diseases [13]. Nonetheless, CI plays a pivotal role during the continuously changing landscape of tumor progression, as it contributes to the control of metabolic plasticity of cancer cells. Indeed, after the first study revealing pathogenic mtDNA mutations in colon cancer [14], a bulk of scientific literature reported the occurrence of somatic mtDNA mutations in nearly every type of human neoplasm. The functional significance of genetic and transcriptional alterations of CI genes, in both nDNA- and mtDNA-encoded subunits, has been investigated, leading to several hypotheses regarding their selection and accumulation in cancer and their role in tumor progression, metastasis formation, and resistance to chemotherapy (Figure 1B,C). In this review, we will summarize the current knowledge on the double-edged role of respiratory CI in cancer biology, focusing on the molecular effects of mtDNA and nDNA mutations and gene expression alteration in CI genes and on the targeting of CI as an adjuvant therapeutic strategy for cancer.

## 2. Mitochondrial DNA Features and Genetics

**Figure 2:**
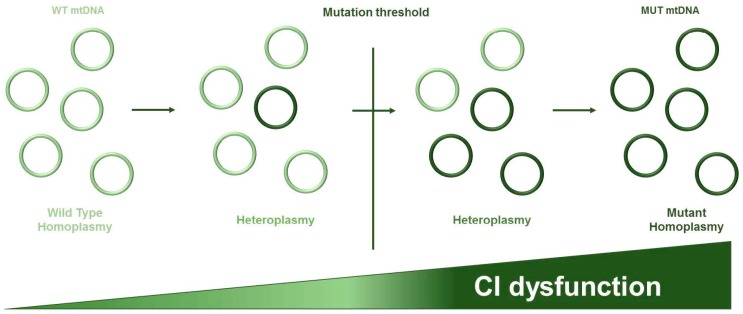
Mitochondrial DNA exists in multiple copies in every cell. The condition of homoplasmy is reached when all mitochondrial DNA (mtDNA) molecules are identical (wild type (WT) or mutant (MUT)), while heteroplasmy is referred to the coexistence of different mitogenomes. Hence, to exert their functional effect mtDNA mutations must be accumulated and surpass a specific mutation threshold that depends on the mutation type and the tissue or cell affected.

Since most CI mutations have been shown to occur in subunits encoded by the mtDNA, a thorough understanding of mitochondrial genetics, which substantially differs from the nuclear one, is necessary to comprehend the origin and functional consequences of such lesions in cancer cells. Human mtDNA is a double-stranded circular molecule of 16,569 bp encoding for 37 genes: 13 genes for respiratory complexes structural subunits and 22 tRNA and 2 rRNA necessary for translation of mitochondrial proteins. One of the peculiar feature of mtDNA is the extreme compact sequence organization. In fact, it lacks repetitive sequences, introns, or intergenic regions, presents with overlapping genes, and transfer RNA (tRNA) genes are used as the signal for cleavage sites of the polycistronic mRNA. The major regulatory non-coding region is the D-loop (displacement loop) which contains the replication origin for the H-strand and the site of transcription from opposing heavy and light strand promoters. A second non-coding region is composed by 30 nucleotides and encompasses the replication origin for the L-strand (reviewed in [16]). Mitochondrial genetics follows its specific rules and differs from Mendelian genetics in three major aspects [17]: (i) maternal inheritance—mitochondria in the zygote derive from the oocyte because after fecundation, all the mitochondria from the spermatocytes are degraded. Hence, mtDNA molecules and germ lines mutations are inherited along the maternal lineage; (ii) heteroplasmy and threshold effect (Figure 2)—mtDNA is polyploid, meaning that a mammalian cell can contain from hundreds to thousands copies of mtDNA. The condition of homoplasmy is reached when all the mtDNA molecules are identical (wild type or mutant), whereas heteroplasmy occurs when different mitogenomes coexist. As a general rule, heteroplasmic mtDNA mutations must trespass a critical load to express their phenotype (threshold effect). This threshold usually ranges between 70% and 90% of mutant mtDNA molecules, but depends of the mutation type and the energy requirements of the affected cell or tissue [18]; (iii) mitotic segregation—the replication of mtDNA and nDNA is not coordinated and the distribution of mitochondria during cell division is casual. For this reason, the mutation load can vary with time in somatic cells.

**Figure 3:**
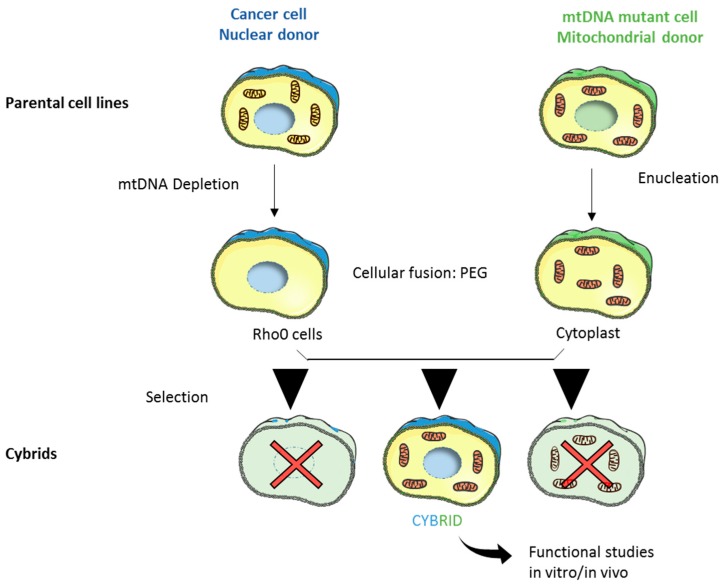
Transmitochondrial cytoplasmic hybrids (cybrids) generation. The original method was developed in Giuseppe Attardi’s laboratory [23] and allows study of the functional effects of mtDNA mutations in an isogenic nuclear background. This three-step technique is based on mitochondria transfer from an enucleated cell line (Rho0, mitochondrial donor) to another nucleated cell (cytoplast, nuclear donor). Cytoplasmic fusion of generated Rho0 cells and cytoplasts using polyethylene glycol (PEG) allows generation of cybrids that will be isolated after an appropriate selection step and used both in vitro and in vivo as cell models to evaluate the impact of mtDNA mutations. This figure has been created by modifying the templates of the empty cell and mitochondria from Servier Medical Art (https://smart.servier.com/smart_image/cell-24/; https://smart.servier.com/smart_image/mitochondria-16/).

The last two peculiar features of mtDNA are particularly important in the context of cancer biology, since somatic mutations occur and accumulate in human tumors. Given the extremely compact organization of mtDNA, which is mostly coding, mutations generally end up affecting protein translation or function, as buffer sequencing such as introns do not occur in mtDNA. Moreover, respiratory CI and complex III are the major sites of ROS production and they have been appointed as causative for the high mutational rate of mtDNA, particularly in the context of somatic mutations generation. However, a recent study demonstrated that the mtDNA mutational pattern found in tumors is more compatible with the physiological mutational mechanism occurring during mtDNA replication rather than other external mutagens, since the mitochondrial DNA polymerase (POLG) is error-prone [19]. Moreover, some germline mutations present at low heteroplasmy in normal tissues may be found enriched in tumors [20,21]. The mechanism through which these mutations are selected and accumulated is still debated. On one hand, it has been shown that mutations found in tumor samples are mostly neutral missense mutations in homoplasmy, whereas severe disruptive mutations are negatively selected and usually found almost exclusively in heteroplasmy [19]. On the other hand, protein-altering mtDNA variants that are present at low frequencies in normal cells preferentially expand under the selective pressure of the tumor microenvironment, suggesting that they may confer a selective advantage to cancer cells [21]. Lastly, it has been proposed that mtDNA mutations are subjected to a relaxed selection, resulting in homoplasmic shift in tumors compatible with a random drift [22]. In this complex scenario, the assessment of the functional role of mtDNA mutations, in particular those in CI subunits genes, is of pivotal importance. A very valuable tool to understand the contribution of specific mtDNA mutations in cancer biology is represented by transmitochondrial cytoplasmic hybrids (cybrids) [23] (Figure 3). This in vitro cell model was created to evaluate the functional role of mtDNA mutations in the context of a different nuclear background. Cybrids are generated from the fusion of a nuclear donor lacking mtDNA (Rho0 cells) and an enucleated mitochondrial donor (cytoplast). Most of the current knowledge about the role of mtDNA mutations in tumorigenic properties of cancer cells and their response to chemotherapy was built taking advantage of functional studies performed on cybrids.

## 3. Relevance of mtDNA Mutations in Complex I Genes in Cancer Progression, Metastasis Formation, and Chemoresistance

A bulk of studies performed in the past 15 years by different groups has aimed at elucidating the contribution of mtDNA mutations in CI genes in every aspect of tumor biology, from carcinogenesis, cancer progression, and metabolic adaptation, to metastasis formation, relapse, and therapy resistance. A major debate regarded the contribution of CI mtDNA mutations in tumor initiation and in the molecular events favoring disease progression. Although a recent study showed an increased risk of developing neoplasia in patients with mitochondrial disorders [24], mtDNA mutations per se are not able to induce carcinogenesis, as genetic alterations in oncogenes and tumor suppressors are still required for tumor initiation. However, certain mtDNA polymorphic variants can be considered risk factors for tumor development. For example, multiple reports linked polymorphism at position 10,398 in *MT-ND3* gene with a higher risk to develop breast and prostate cancer in different populations [25,26,27,28,29]. Forced overexpression of mutant forms of *MT-ND5* or *MT-ND2* with missense mutations in cancer cells carrying wild type mtDNA increases ROSproduction, confers a growth advantage and stimulates aerobic glycolysis through hypoxia-inducible factor 1α (HIF1α) [30,31,32]. Increased oxidative stress and activation of Akt pathway were also involved in promotion of tumor aggressiveness of lung carcinoma cybrids carrying *MT-ND6* mutations [33]. In this work, the occurrence of such mutations correlates with the pathological grade, tumor stage, lymph node metastasis, and with shorter survival rate, implying a negative contribution of such mutations to disease prognosis [33]. Moreover, the occurrence of homoplasmic missense mutations as m.11778G>A/*MT-ND4* and m.14484T>C/*MT-ND6* that are well known to induce a mild CI deficiency and to stimulate ROS production have been shown to enhance tumorigenesis of osteosarcoma cybrids [34]. The bioenergetic features of these mutations have been extensively studied as they are primary pathogenic mutations for Leber’s hereditary optic neuropathy (LHON), the most common mitochondrial disorder [35]. Interestingly, the third primary LHON mutation m.3460G˃A/*MT-ND1*, known to be the most severe in terms of bioenergetic defects and clinical penetrance, has a very mild effect on tumor growth and can be considered neutral [34,36]. To further complicate this scenario, the heteroplasmic frameshift m.12417insA/*MT-ND5* mutation was found to exert a pro-tumorigenic effect in vitro and in vivo stimulating ROS production which in turn leads to the activation of Akt kinase [37,38]. However, when this mutation reaches homoplasmy, the consequent severe CI defect prevents tumor growth stimulating the apoptotic pathway [37], clearly indicating that the impact on tumor progression depends on the mutation load and on its bioenergetic consequences. In this context, our group demonstrated that homoplasmic disruptive mutations in mtDNA-encoded CI, such as the m.3571insC/*MT-ND1*, hampered tumor growth of cancer cells deriving from different tissues [36,39]. Beside the evident energy crisis, we found that the lack of CI was associated with complex metabolic alterations, including the imbalance of α-ketoglutarate (αKG)/succinate ratio [36,39]. These metabolites are allosteric regulators of prolyl hydroxylases (PHDs), αKG–dependent enzymes responsible for the hydroxylation of several targets, including HIF1α [40]. Imbalance of αKG/succinate activates PHDs even in hypoxia, leading to hydroxylation and continuous degradation of HIF1α, thus preventing hypoxic adaptation and the generation of a Warburg transcriptional profile [36,39,41]. The outcome of such inability of CI-defective cancer cells to adapt to the selective pressures of tumor microenvironment was the block of tumor growth in vivo. The lack of CI due to the m.3571insC/*MT-ND1* mutation induced the accumulation of structurally altered mitochondria, similar to what observed in oncocytomas, a subset of epithelial neoplasms with a generally indolent behavior [42]. The presence of homoplasmic disruptive mtDNA mutations in CI genes triggers the oncocytic phenotype and confines aggressive cancers, such as osteosarcoma, into a benign state of quiescence typical of oncocytomas, while the same mutations in heteroplasmy do not affect tumor progression [42]. Our findings allowed us to highlight for the first time the *oncojanus* nature of mtDNA mutations in respiratory CI genes, based on their effect on CI integrity and on the subsequent alterations on the overall metabolic status of the cell, which is a function of their heteroplasmic load as well [36,39,41]. In support of our hypothesis, a recent analysis on mtDNA mutational burden in breast cancer showed that patients with the highest accumulation of somatic mutations present with better overall survival [43].

Some studies have also investigated the possible role of mtDNA mutations in CI genes in metastasis formation. In 2008, Hayashi’s group reported that mouse mtDNA carrying the two mutations m.13997G>A and m.13885insC in *mt-nd6* was able to confer high metastatic potential when transferred into low metastatic cancer cells [44]. The authors showed that this phenotype was caused by reduction of CI activity and ROS overproduction, and that it can be reversed by treatment with antioxidants. Increased levels of HIF1α correlated with ROS production and were thought to be responsible for the more aggressive phenotype of mutated cells, albeit this finding was not reported in the same paper for the frameshift mutation in *mt-nd6*, but only for the missense one. The substitution m.13997G>A/*mt-nd6* was also associated with increased metastatic potential and B-lymphomas development in transmitochondrial mice, but only in the presence of a favorable specific nuclear background [45,46]. Interestingly, transfer of mtDNA carrying specific missense mutations was able to boost metastasis formation also in the context of breast cancer [47,48]. A set of mtDNA mutations predicted to affect CI function was found in association with the occurrence of distant metastases in non-small cell lung carcinoma (NSCLC) and colon cancers patients [49]. Overall, these works suggest that missense mtDNA mutations may promote invasiveness and metastasis formation. This is in contrast with the finding that acquisition of mtDNA, and the consequent OXPHOS functionality, is necessary for escape from dormancy of metastatic breast cancer cells [50] and that highly metastatic cancer cells showed an enhanced OXPHOS function [51]. Hence, the role of mtDNA mutations in CI genes in promoting invasiveness is far from being elucidated. For example, a recent work showed that certain mtDNA landscapes rather than specific mtDNA mutations identify metastatic cells, as these mitogenomes stimulate the mitochondrial unfolded protein response, promoting metastasis formation [52].

Lastly, mitochondria have been recently recognized as important players in response to chemotherapy (reviewed in [53]). The first report about the contribution of mtDNA in resistance to chemotherapy showed that HeLa cybrids carrying several mtDNA mutations, including m.10970T>C/*MT-ND4* and m.10176G>A/*MT-ND3*, were resistant to 5-fluorouracil and cisplatin in vitro and in vivo [54]. Resistance to cisplatin has also been shown in A549 lung carcinoma cells in which the m.4587T>C/*MT-ND2* mutation shifted toward homoplasmy accompanied by increased mitochondrial biogenesis [55]. The suppression of mitochondrial biogenesis restores sensitivity to platinum, but the molecular bases of such phenomenon are still unknown. In this context, we showed that exposure to cisplatin of cancer cells of gynecological origin induced the occurrence of m.13828C>T/*MT-ND5* and the m.8156G>T/*MT-CO2* mutations [56]. Such mutations induce a respiration-deficient phenotype and reduce proliferative and tumorigenic potential, in terms of migratory and invasive capacity. Moreover, the bioenergetic defect also hampers cytoskeletal organization, in particular filamentous tubulin, which is the main target of paclitaxel. Indeed, mtDNA mutations are positively selected and quickly shift to homoplasmy in paclitaxel-resistant clones, and such resistance is acquired by osteosarcoma cybrids when mtDNA with homoplasmic mutations is transferred into this nuclear background, highlighting the causative role of mtDNA mutations in the acquisition of resistance to paclitaxel [56]. This study is supported by our previous in vivo observation of a residual mass from a patient with ovarian cancer treated with carboplatin and paclitaxel. The post-chemotherapy paclitaxel-resistant specimen acquired the nearly homoplasmic disruptive m.10875T>C/*MT-ND4* mutation and showed an oncocytic phenotype [57], resulting in a more quiescent, non-invasive, and low-proliferative neoplasm and reflecting again the *oncojanus* nature of mtDNA mutations in CI genes.

## 4. Emerging Role of nDNA-Encoded Subunits and Assembly Factors of Complex I in Cancer

Several studies showed the altered expression of some nDNA-encoded CI subunits in different tumor types. Lower levels of NDUFS1, NDUFS3, NDUFA1, NDUFA4, and NDUFB6 subunits correlated with poor prognosis and may be considered as prognostic markers for survival and disease outcome [58,59,60,61,62]. The analysis of whole exomes from melanoma patients present in TCGA (The Cancer Genome Atlas; https://cancergenome.nih.gov/) allowed identification of a recurrent mutation in the promoter region of NDUFB9 which was predicted to disrupt a highly conserved SP1/KLF transcription factor binding motif. The occurrence of this mutation reduced transcriptional activity, suggesting it may lead to decreased NDUFB9 levels in melanomas [63]. Moreover, knockdown of NDUFB9, NDUFS3, NDUFA13, and NDUFV1 and the consequent reduction of CI activity correlated with increased invasiveness of breast cancer cells [64,65,66], although studies in which these genes are knocked out completely, and CI expression/assembly is abolished are currently lacking, and may be relevant in light of the importance of a functional threshold for a phenotypic effect that this enzyme displays. Reduced CI activity upon NDUFV1 downregulation has shown to decrease the NAD^+^/NADH ratio, promoting tumor metastasis in vivo, while treating cancer cells with NAD^+^ precursors prevents this phenotype by inducing autophagy and downregulating the Akt and mammalian Target Of Rapamycin Complex 1 (mTORC1) pathway, in a ROS independent fashion [66]. On the other hand, downregulation of NDUFB9 was found to stimulate mitochondrial ROS levels leading to the activation of the Akt/mTOR signaling pathway, which subsequently promoted invasion and induced the epithelial-mesenchymal transition (EMT) [64]. Lower NDUFS3 and NDUFA13 levels were also related to increased ROS levels and epithelial–mesenchymal transition (EMT) induction [65]. In particular, the CI accessory subunit NDUFA13, also known as Gene associated with Retinoic-Interferon-induced Mortality 19 (GRIM-19), has been widely investigated for its role in tumor progression and metastasis formation [67]. This protein was first identified as a regulator of apoptosis with nuclear localization [68], but very soon it became clear that it was an accessory subunit of CI [69]. Loss of NDUFA13 completely abolishes CI activity and stimulates the Warburg effect, triggering the expression of glycolytic genes [70,71]. Interestingly, the deletion of a single copy of the NDUFA13 gene was found to be sufficient to promote carcinogenesis of invasive squamous cell carcinomas [71]. Besides its role as CI structural subunit, NDUFA13 has been shown to suppress the activity of Signal Transducer and Activator of Transcription 3 (STAT3) by direct interaction [72]. NDUFA13 has been found to act as a tumor suppressor by keeping STAT3 in its inactive state and preventing oncogenic transformation, cell survival and proliferation, migration, and EMT [73,74]. Indeed, NDUFA13 has been found downregulated in different tumors including hepatocellular, cervical, colorectal, renal cell carcinoma, and breast cancer compared to their respective normal tissues [75,76,77,78,79]. Moreover, heterozygous missense mutations in —GRIM-19 were found in few cases of oncocytic thyroid carcinomas, but not in non-oncocytic tumors or in peripheral blood from control individuals [80]. The authors hypothesized that two of these mutations (G264C and G593C), being located in a phylogenetically conserved region, may alter the protein expression or function inducing CI dysfunction. However, it is important to note that these mutations have been found exclusively in oncocytic carcinomas and not in other thyroid tumors, indicating that the hypothetical CI dysfunction may be responsible for the accumulation of dysfunctional mitochondria rather than being involved in the tumorigenesis process. In this context, functional studies that would help to elucidate the role of these mutations are still missing. Overall, these works support the idea that a reduced activity of CI caused by downregulation or mutations in nDNA-encoded CI subunits promotes tumorigenesis and cancer cell invasiveness. However, conflicting reports are present in the current literature. For instance, NDUFS3 was found preferentially upregulated in hypoxic/necrotic areas in invasive ductal carcinoma and can be considered a prognostic marker for such tumors [81]. Another clear example is represented by the opposite expression of NDUFS1 and NDUFS8 in NSCLC [82]. In this study, a possible *oncojanus* role of nuclear CI genes is proposed, as an overall poor prognosis in a big cohort of NSCLC patients was associated with the combination of low NDUFS1 and high NDUFS8 expression levels, while the opposite phenotype (high NDUFS1 and low NDUFS8) seemed to have a protective effect. The authors proposed a possible explanation of this two-sided effect in the multi-step process of CI biogenesis, in which subunits belonging to the N-module, as NDUFS1 or NDUFV1, are incorporated in the last step, while subunits belonging to the Q-module, as NDUFS8, are involved in the early steps of CI assembly [2]. In this frame, reduced expression of N-module subunits may have a mild impact on CI function, while decreased levels of those belonging to the Q-module would induce a severe dysfunction leading to opposite effects in terms of tumor aggressiveness [82], similar to what we here propose for the functional effects of mtDNA mutations. Moreover, benign oncocytomas show reduced levels of nDNA-encoded CI subunits, probably due to the lack of mtDNA-encoded subunits [83,84,85,86]. Overall, an increasing amount of reports showed that the expression of nDNA-encoded CI genes may influence cancer biology, but very few studies investigated their molecular and biochemical consequences. Nonetheless, the *oncojanus* nature of CI has already emerged, pointing out the need for further efforts to elucidate the role of nDNA-encoded subunits.

## 5. Complex I as a Target for Anti-Cancer Therapy?

Based on these premises, it is clear that a possibility to integrate the already existing therapeutic protocols with other drugs that severely affect CI function is an intriguing hypothesis. However, the detrimental effect of ROS overproduction caused by CI inhibition and the possibility of adverse effects due to the blocking of the function of a ubiquitous and essential enzyme must be taken into account. Classical CI inhibitors (rotenone, piericidin A, and capsaicin) were found to selectively decrease tumorigenic potential under metabolic stress conditions in different cancer cell lines [87] (Table 1). The use of rotenone and piericidin A seems to remain confined into preclinical studies since these inhibitors blocked electron transfer from Fe–S clusters to the ubiquinone, favoring ROS production [88]. 

Biguanides metformin and phenformin have recently drawn attention for their role as potential anticancer CI inhibitors (Table 1). In particular, metformin has been widely used for many years for treatment of type II diabetes and several epidemiological studies highlighted the beneficial effect of metformin in oncologic patients, in terms of reduced risk to develop disease and better prognosis [89,90,91]. On these bases, several clinical trials on metformin in different neoplasias have been undertaken in the past few years. However, the mechanisms through which metformin prevents tumor progression are not fully understood and most likely depend on the combination of intracellular and systemic effects of this drug. Nonetheless, other newly discovered CI inhibitors have been recently found to prevent tumor progression, namely BAY 87-2243 and AG311 (Table 1) [92,93]. These small molecules share some molecular effects related to CI inhibition that may explain their anticancer properties. First, they are more effective under glucose starvation, suggesting that cancer cells mostly rely on glycolysis to survive upon CI inhibition. Accordingly, when glucose concentrations are limited, treated cells experience a profound ATP depletion leading to cell death [93,94,95]. Such energetic crisis triggers the activation of the cell energy sensor adenosine monophosphate (AMP)-activated kinase (AMPK), which inhibits mTORC1 signaling and cell cycle progression and stimulates catabolic reactions to restore the AMP/ATP ratio. In this context, AMPK seems to play an anti-tumorigenic role, although this mechanism cannot exclusively explain the anti-proliferative effect of CI inhibitors as many cancers unable to activate AMPK are still susceptible to these drugs [96]. We found activated AMPK in CI-defective models carrying disruptive mtDNA mutations in CI genes and demonstrated that the activation of this cellular energetic sensor was related to the metabolic adaptation of cells when CI was missing (i.e., activation of mitochondrial biogenesis, fatty acid oxidation, and glycolysis stimulation), rather than being a major player in the block of tumor growth [36]. Concordantly with the demonstrated effects of disruptive CI mutations, metformin, BAY 87-2243, and AG311 also share the ability to destabilize HIF1α and prevent hypoxic adaptation [92,93,94]. Increased activation of PHD may explain the degradation of HIF1α under hypoxic conditions upon CI inhibition. This can occur through the previously described mechanism mediated by αKG accumulation and allosteric activation of PHD (pseudonormoxia), and/or by the fact that CI inhibition reduces the intracellular oxygen consumption leading to a condition of intracellular normoxia even when the extracellular oxygen tension is low, as recently described [97]. Metformin, but not BAY 87-2243 and AG311, may also have the additional beneficial effect to reduce ROS production, which are known to stimulate HIF1α transcription in an Akt-mediated fashion independently from oxygen levels [40].

Another promising drug is fenofibrate, a compound belonging to the fibrates family used for the therapy of hypercholesterolemia and hypertriglyceridemia and known to reversibly inhibit CI [98]. CI inhibition by fenofibrate induces apoptotic cell death, although cancer cells attempt to respond to the energetic deficit by stimulating autophagy to sustain cell proliferation [99]. Lastly, several other synthetic and natural compounds have been recently identified as CI inhibitors with a potential activity on cancer cells as anti-proliferative drugs (Table 1). All these molecules are at very preliminary stages of investigation, but they are still very promising as they target the same pathways identified through ablation of CI by genetic studies, namely adaptation to hypoxia and induction of apoptosis. The continuously growing number of reports of the anti-proliferative activity of CI inhibitors strongly corroborates the hypothesis that the complete block of this enzyme is detrimental for tumor progression and opens the door to new possible therapeutic approaches for solid tumors, as mitochondrial function in general and adaptation to hypoxia are necessary for tumor progression. Nonetheless, other efforts are necessary to disentangle the molecular mechanisms activated in response to CI inhibition and to identify compounds that are safe enough to act selectively on cancer cells without drastic side effects.

## 6. Concluding Remarks

**Figure 4:**
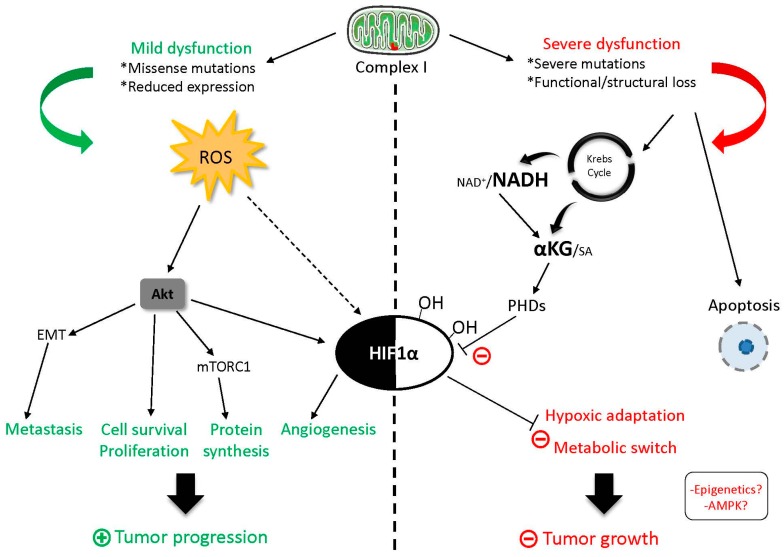
The *oncojanus* effect of CI encoding genes on tumorigenesis. Mild functional alterations of CI cause mitochondrial ROS overproduction and subsequent activation of the oncogenic Akt/mTORC1 (mammalian Target Of Rapamycin Complex 1) pathway that leads to cell proliferation and survival, epithelial–mesenchymal transition (EMT), and HIF1α stabilization, thus favoring tumor progression. Conversely, severe CI dysfunction provokes the accumulation of tricarboxylic acids (TCA) cycle products NADH and α-ketoglutarate (KG), which impedes Hypoxia Inducible Factor (HIF) 1α stabilization, hypoxic adaptation, ultimately arresting tumor growth. Moreover, the energetic crisis stimulates apoptotic cell death.

In the past years, an increasing number of studies have highlighted the pivotal role of the mitochondrial OXPHOS system and, in particular, of CI in all aspects of cancer biology. The initial hypothesis made by Otto Warburg suggested that mitochondrial respiratory function must be suppressed in cancer cells as they perform aerobic glycolysis, and this may drive carcinogenesis. However, recent studies clearly demonstrate that mitochondrial respiration is necessary for tumor progression and escape from dormancy of cancer cells [50,111,112]. Similarly, the first studies reported that CI impairment was associated with increased tumorigenic properties of cancer cells, but now it has been found that the severity of CI dysfunction strongly influences tumor progression, metastasis formation, and resistance to certain chemotherapeutics (Figure 4). Hence, respiratory CI can be considered the prototype of *oncojanus*. It is interesting to note that mutations in mtDNA genes encoding for CI subunits are frequently found in cancers, where they may contribute to tumor metabolism and, in turn, tumorigenic properties of cancer cells. However, these mutations are usually passenger events and only mutations with neutral/mild impact on CI function are accumulated in tumors [19]. The only remarkable exception is represented by oncocytomas, where disruptive CI mutations in mtDNA induce a metabolic short-circuit, leading to an almost benign phenotype [42]. MtDNA peculiarities favor the occurrence and the selection of somatic mutations, while nDNA-encoded CI genes are usually not affected by genetic lesions, since the consequent CI dysfunction would be transmitted to every daughter cell, leading to a profound and generalized metabolic alteration. Indeed, deregulation of the expression of CI subunits encoded by nDNA has been observed in tumors. The functional role of these alterations is still unknown and deserves in-depth investigations. Moreover, the current knowledge on the contribution of CI dysfunction to resistance to chemotherapy is still very poor, opening a still unexplored research area. Lastly, it is important to note that different metabolic signals derived from CI dysfunction mainly converge on two molecular players, namely the mTORC1 and HIF1α (Figure 4). Multiple metabolic signals point to the modulation of these factors. On one hand, severe CI dysfunction reduces energy charge and impedes mTORC1 activation, blocking protein synthesis and cell proliferation, while NADH accumulation prevents the stabilization of HIF1α. Conversely, inhibition of CI function promotes ROS production, thus triggering Akt activation, which in turn stimulates mTORC1 activation and HIF1α transcription (Figure 4).

As targeting CI is gaining momentum as a possible adjuvant therapeutic strategy for cancer, it is of pivotal importance to disentangle the metabolic consequence and the molecular mechanisms triggered by CI ablation or inhibition. Moreover, it is important to note that, as every pharmacological approach, CI inhibition may also be subject to the induction of resistance and that the unmatched plasticity of cancer cells may stimulate some still unknown salvage pathways, leading to tumor relapse. In the coming years, it would be desirable to investigate these aspects in order to develop more effective and less toxic therapies for human cancers.

## Figures and Tables

**Table 1:** Mitochondrial CI inhibitors with anti-cancer proprieties.

Inhibitor	Mechanism of Action	Cellular Model	References
Rotenone	Induces cell death under starvation in vitro Increases ROS production in vitro	Breast cancer cells, bovine heart mitochondria	[87,88]
Piericidin A	Induces cell death under starvation in vitro Increases ROS production in vitro	Breast, pancreatic and lung cancer cells, bovine heart mitochondria	[87]
Capsaicin	Induces cell death under starvation in vitro	Breast cancer cells	[87]
Metformin	Induces cell death under starvation in vitro Promotes AMPK phosphorylation Induces HIF1α destabilization under hypoxic conditions	Colon, lung, breast, cervical, osteosarcoma, oral carcinoma cancer cells	[94,96,100,101,102]
Phenformin	Induces cell death under starvation in vitro Promotes AMPK phosphorylation Induces HIF1α destabilization under hypoxic conditions	Colon and breast cancer cells and xenografts	[94,103,104,105]
BAY 87-2243	Induces cell death under starvation in vitro Decreases tumor growth in vivo Promotes AMPK phosphorylation Induces HIF1α destabilization under hypoxic conditionsIncreases intracellular ROS levels	Lung cancer and melanoma cells and xenografts	[92,95]
AG311	Induces cell death under starvation in vitro Decreases tumor growth in vivo Promotes AMPK phosphorylation Induces HIF1α destabilization under hypoxic conditions Induces mitochondrial superoxide production	Breast cancer cells and xenografts	[93,106]
Fenofibrate	Induces cell death in vitro	Glioblastoma cells	[99]
JCI-20679	Induces cell death in vitro	A panel of 39 human cancer cell lines	[107]
Celastrol	Promotes ROS production and apoptosis in vitro	Lung cancer and hepatocellular carcinoma cells	[108]
Kalkitoxin	Induces cell death in vitro Induces HIF1α destabilization under hypoxic conditions	Neuroblastoma, breast and colon cancer cells	[109]
Lehualide B	Induces cell death in vitro	Multiple myeloma cells	[110]

AMPK: Adenosine monophosphate (AMP)-activated kinase; HIF1α: Hypoxia-inducible factor 1α; ROS: Reactive oxygen species.

## References

[1] Milenkovic D., Blaza J.N., Larsson N.-G., Hirst J. (2017). The enigma of the respiratory chain supercomplex. Cell Metab..

[2] Guerrero-Castillo S., Baertling F., Kownatzki D., Wessels H.J., Arnold S., Brandt U., Nijtmans L. (2017). The assembly pathway of mitochondrial respiratory chain complex I. Cell Metab..

[3] Sánchez-Caballero L., Guerrero-Castillo S., Nijtmans L. (2016). Unraveling the complexity of mitochondrial complex I assembly: A dynamic process. Biochim. Biophys. Acta.

[4] Hofhaus G., Weiss H., Leonard K. (1991). Electron microscopic analysis of the peripheral and membrane parts of mitochondrial NADH dehydrogenase (complex I). J. Mol. Biol..

[5] Ragan C.I., Hatefi Y. (1986). Isolation of the iron–sulfur-containing polypeptides of NADH:Oxidoreductase ubiquinone. Methods Enzymol..

[6] Vinogradov A.D., Grivennikova V.G. (2016). Oxidation of NADH and ROS production by respiratory complex I. Biochim. Biophys. Acta.

[7] Iommarini L., Calvaruso M.A., Kurelac I., Gasparre G., Porcelli A.M. (2013). Complex I impairment in mitochondrial diseases and cancer: Parallel roads leading to different outcomes. Int. J. Biochem. Cell Biol..

[8] DiMauro S., Schon E.A., Carelli V., Hirano M. (2013). The clinical maze of mitochondrial neurology. Nat. Rev. Neurol..

[9] Blandini F., Nappi G., Greenamyre J.T. (1998). Quantitative study of mitochondrial complex I in platelets of parkinsonian patients. Mov. Disord. Off. J. Mov. Disord. Soc..

[10] Giordano L., Deceglie S., d’Adamo P., Valentino M.L., La Morgia C., Fracasso F., Roberti M., Cappellari M., Petrosillo G., Ciaravolo S. (2015). Cigarette toxicity triggers Leber’s hereditary optic neuropathy by affecting mtDNA copy number, oxidative phosphorylation and ROS detoxification pathways. Cell Death Dis..

[11] Ghelli A., Porcelli A.M., Zanna C., Vidoni S., Mattioli S., Barbieri A., Iommarini L., Pala M., Achilli A., Torroni A. (2009). The background of mitochondrial DNA haplogroup J increases the sensitivity of Leber’s hereditary optic neuropathy cells to 2,5-hexanedione toxicity. PLoS ONE.

[12] Rodenburg R.J. (2016). Mitochondrial complex I-linked disease. Biochim. Biophys. Acta.

[13] Giannoccaro M.P., La Morgia C., Rizzo G., Carelli V. (2017). Mitochondrial DNA and primary mitochondrial dysfunction in Parkinson’s disease. Mov. Disord. Off. J. Mov. Disord. Soc..

[14] Polyak K., Li Y., Zhu H., Lengauer C., Willson J.K., Markowitz S.D., Trush M.A., Kinzler K.W., Vogelstein B. (1998). Somatic mutations of the mitochondrial genome in human colorectal tumours. Nat. Genet..

[15] Fiedorczuk K., Letts J.A., Degliesposti G., Kaszuba K., Skehel M., Sazanov L.A. (2016). Atomic structure of the entire mammalian mitochondrial complex I. Nature.

[16] Bestwick M.L., Shadel G.S. (2013). Accessorizing the human mitochondrial transcription machinery. Trends Biochem. Sci..

[17] Schon E.A., DiMauro S., Hirano M. (2012). Human mitochondrial DNA: Roles of inherited and somatic mutations. Nat. Rev. Genet..

[18] Rossignol R., Faustin B., Rocher C., Malgat M., Mazat J.-P., Letellier T. (2003). Mitochondrial threshold effects. Biochem. J..

[19] Ju Y.S., Alexandrov L.B., Gerstung M., Martincorena I., Nik-Zainal S., Ramakrishna M., Davies H.R., Papaemmanuil E., Gundem G., Shlien A. (2014). Origins and functional consequences of somatic mitochondrial DNA mutations in human cancer. eLife.

[20] Gasparre G., Iommarini L., Porcelli A.M., Lang M., Ferri G.G., Kurelac I., Zuntini R., Mariani E., Pennisi L.F., Pasquini E. (2009). An inherited mitochondrial DNA disruptive mutation shifts to homoplasmy in oncocytic tumor cells. Hum. Mutat..

[21] Grandhi S., Bosworth C., Maddox W., Sensiba C., Akhavanfard S., Ni Y., LaFramboise T. (2017). Heteroplasmic shifts in tumor mitochondrial genomes reveal tissue-specific signals of relaxed and positive selection. Hum. Mol. Genet..

[22] Coller H.A., Khrapko K., Bodyak N.D., Nekhaeva E., Herrero-Jimenez P., Thilly W.G. (2001). High frequency of homoplasmic mitochondrial DNA mutations in human tumors can be explained without selection. Nat. Genet..

[23] King M.P., Attardi G. (1989). Human cells lacking mtDNA: Repopulation with exogenous mitochondria by complementation. Science.

[24] Finsterer J., Frank M. (2016). Prevalence of neoplasms in definite and probable mitochondrial disorders. Mitochondrion.

[25] Canter J.A., Kallianpur A.R., Parl F.F., Millikan R.C. (2005). Mitochondrial DNA G10398A polymorphism and invasive breast cancer in African-American women. Cancer Res..

[26] Bai R.K., Leal S.M., Covarrubias D., Liu A., Wong L.J.C. (2007). Mitochondrial genetic background modifies breast cancer risk. Cancer Res..

[27] Darvishi K., Sharma S., Bhat A.K., Rai E., Bamezai R.N.K. (2007). Mitochondrial DNA G10398A polymorphism imparts maternal haplogroup N a risk for breast and esophageal cancer. Cancer Lett..

[28] Czarnecka A.M., Krawczyk T., Zdrozny M., Lubiński J., Arnold R.S., Kukwa W., Scińska A., Golik P., Bartnik E., Petros J.A. (2010). Mitochondrial NADH-dehydrogenase subunit 3 (ND3) polymorphism (A10398G) and sporadic breast cancer in Poland. Breast Cancer Res. Treat..

[29] Mims M.P., Hayes T.G., Zheng S., Leal S.M., Frolov A., Ittmann M.M., Wheeler T.M., Prchal J.T. (2006). Mitochondrial DNA G10398A polymorphism and invasive breast cancer in African-American women. Cancer Res..

[30] Dasgupta S., Soudry E., Mukhopadhyay N., Shao C., Yee J., Lam S., Lam W., Zhang W., Gazdar A.F., Fisher P.B. (2012). Mitochondrial DNA mutations in respiratory complex-I in never-smoker lung cancer patients contribute to lung cancer progression and associated with EGFR gene mutation. J. Cell. Physiol..

[31] Sun W., Zhou S., Chang S.S., McFate T., Verma A., Califano J.A. (2009). Mitochondrial mutations contribute to HIF1α accumulation via increased reactive oxygen species and up-regulated pyruvate dehydrogenease kinase 2 in head and neck squamous cell carcinoma. Clin. Cancer Res. Off. J. Am. Assoc. Cancer Res..

[32] Zhou S., Kachhap S., Sun W., Wu G., Chuang A., Poeta L., Grumbine L., Mithani S.K., Chatterjee A., Koch W. (2007). Frequency and phenotypic implications of mitochondrial DNA mutations in human squamous cell cancers of the head and neck. Proc. Natl. Acad. Sci. USA.

[33] Yuan Y., Wang W., Li H., Yu Y., Tao J., Huang S., Zeng Z. (2015). Nonsense and missense mutation of mitochondrial ND6 gene promotes cell migration and invasion in human lung adenocarcinoma. BMC Cancer.

[34] Cruz-Bermúdez A., Vallejo C.G., Vicente-Blanco R.J., Gallardo M.E., Fernández-Moreno M.Á., Quintanilla M., Garesse R. (2015). Enhanced tumorigenicity by mitochondrial DNA mild mutations. Oncotarget.

[35] Carelli V., La Morgia C., Iommarini L., Carroccia R., Mattiazzi M., Sangiorgi S., Farne’ S., Maresca A., Foscarini B., Lanzi L. (2007). Mitochondrial optic neuropathies: How two genomes may kill the same cell type?. Biosci. Rep..

[36] Iommarini L., Kurelac I., Capristo M., Calvaruso M.A., Giorgio V., Bergamini C., Ghelli A., Nanni P., De Giovanni C., Carelli V. (2014). Different mtDNA mutations modify tumor progression in dependence of the degree of respiratory complex I impairment. Hum. Mol. Genet..

[37] Park J.S., Sharma L.K., Li H., Xiang R., Holstein D., Wu J., Lechleiter J., Naylor S.L., Deng J.J., Lu J. (2009). A heteroplasmic, not homoplasmic, mitochondrial DNA mutation promotes tumorigenesis via alteration in reactive oxygen species generation and apoptosis. Hum. Mol. Genet..

[38] Sharma L.K., Fang H., Liu J., Vartak R., Deng J., Bai Y. (2011). Mitochondrial respiratory complex I dysfunction promotes tumorigenesis through ROS alteration and AKT activation. Hum. Mol. Genet..

[39] Gasparre G., Kurelac I., Capristo M., Iommarini L., Ghelli A., Ceccarelli C., Nicoletti G., Nanni P., De Giovanni C., Scotlandi K. (2011). A mutation threshold distinguishes the antitumorigenic effects of the mitochondrial gene *MTND1*, an oncojanus function. Cancer Res..

[40] Iommarini L., Porcelli A.M., Gasparre G., Kurelac I. (2017). Non-canonical mechanisms regulating hypoxia-inducible factor 1 α in cancer. Front. Oncol..

[41] Calabrese C., Iommarini L., Kurelac I., Calvaruso M.A., Capristo M., Lollini P.L., Nanni P., Bergamini C., Nicoletti G., Giovanni C.D. (2013). Respiratory complex I is essential to induce a Warburg profile in mitochondria-defective tumor cells. Cancer Metab..

[42] De Luise M., Girolimetti G., Okere B., Porcelli A.M., Kurelac I., Gasparre G. (2017). Molecular and metabolic features of oncocytomas: Seeking the blueprints of indolent cancers. Biochim. Biophys. Acta.

[43] McMahon S., LaFramboise T. (2014). Mutational patterns in the breast cancer mitochondrial genome, with clinical correlates. Carcinogenesis.

[44] Ishikawa K., Takenaga K., Akimoto M., Koshikawa N., Yamaguchi A., Imanishi H., Nakada K., Honma Y., Hayashi J.I. (2008). ROS-generating mitochondrial DNA mutations can regulate tumor cell metastasis. Science.

[45] Hashizume O., Shimizu A., Yokota M., Sugiyama A., Nakada K., Miyoshi H., Itami M., Ohira M., Nagase H., Takenaga K. (2012). Specific mitochondrial DNA mutation in mice regulates diabetes and lymphoma development. Proc. Natl. Acad. Sci. USA.

[46] Hashizume O., Yamanashi H., Taketo M.M., Nakada K., Hayashi J.-I. (2015). A specific nuclear DNA background is required for high frequency lymphoma development in transmitochondrial mice with G13997A mtDNA. PLoS ONE.

[47] Kulawiec M., Owens K.M., Singh K.K. (2009). mtDNA G10398A variant in African-American women with breast cancer provides resistance to apoptosis and promotes metastasis in mice. J. Hum. Genet..

[48] Imanishi H., Hattori K., Wada R., Ishikawa K., Fukuda S., Takenaga K., Nakada K., Hayashi J. (2011). Mitochondrial DNA mutations regulate metastasis of human breast cancer cells. PLoS ONE.

[49] Koshikawa N., Akimoto M., Hayashi J.I., Nagase H., Takenaga K. (2017). Association of predicted pathogenic mutations in mitochondrial ND genes with distant metastasis in NSCLC and colon cancer. Sci. Rep..

[50] Sansone P., Savini C., Kurelac I., Chang Q., Amato L.B., Strillacci A., Stepanova A., Iommarini L., Mastroleo C., Daly L. (2017). Packaging and transfer of mitochondrial DNA via exosomes regulate escape from dormancy in hormonal therapy-resistant breast cancer. Proc. Natl. Acad. Sci. USA.

[51] Porporato P.E., Payen V.L., Pérez-Escuredo J., De Saedeleer C.J., Danhier P., Copetti T., Dhup S., Tardy M., Vazeille T., Bouzin C. (2014). A mitochondrial switch promotes tumor metastasis. Cell Rep..

[52] Kenny T.C., Hart P., Ragazzi M., Sersinghe M., Chipuk J., Sagar M.A.K., Eliceiri K.W., LaFramboise T., Grandhi S., Santos J. (2017). Selected mitochondrial DNA landscapes activate the SIRT3 axis of the UPRmtto promote metastasis. Oncogene.

[53] Guerra F., Arbini A.A., Moro L. (2017). Mitochondria and cancer chemoresistance. Biochim. Biophys. Acta.

[54] Mizutani S., Miyato Y., Shidara Y., Asoh S., Tokunaga A., Tajiri T., Ohta S. (2009). Mutations in the mitochondrial genome confer resistance of cancer cells to anticancer drugs. Cancer Sci..

[55] Yao Z., Jones A.W.E., Fassone E., Sweeney M.G., Lebiedzinska M., Suski J.M., Wieckowski M.R., Tajeddine N., Hargreaves I.P., Yasukawa T. (2013). PGC-1β mediates adaptive chemoresistance associated with mitochondrial DNA mutations. Oncogene.

[56] Girolimetti G., Guerra F., Iommarini L., Kurelac I., Vergara D., Maffia M., Vidone M., Amato L.B., Leone G., Dusi S. (2017). Platinum-induced mitochondrial DNA mutations confer lower sensitivity to paclitaxel by impairing tubulin cytoskeletal organization. Hum. Mol. Genet..

[57] Guerra F., Perrone A.M., Kurelac I., Santini D., Ceccarelli C., Cricca M., Zamagni C., De Iaco P., Gasparre G. (2012). Mitochondrial DNA mutation in serous ovarian cancer: Implications for mitochondria-coded genes in chemoresistance. J. Clin. Oncol. Off. J. Am. Soc. Clin. Oncol..

[58] Ellinger J., Poss M., Brüggemann M., Gromes A., Schmidt D., Ellinger N., Tolkach Y., Dietrich D., Kristiansen G., Müller S.C. (2017). Systematic expression analysis of mitochondrial complex I identifies NDUFS1 as a biomarker in clear-cell renal-cell carcinoma. Clin. Genitourin. Cancer.

[59] Wang P., Cheng X., Fu Z., Zhou C., Lu W., Xie X. (2013). Reduced expression of NDUFS3 and its clinical significance in serous ovarian cancer. Int. J. Gynecol. Cancer Off. J. Int. Gynecol. Cancer Soc..

[60] Mamelak A.J., Kowalski J., Murphy K., Yadava N., Zahurak M., Kouba D.J., Howell B.G., Tzu J., Cummins D.L., Liégeois N.J. (2005). Downregulation of NDUFA1 and other oxidative phosphorylation-related genes is a consistent feature of basal cell carcinoma. Exp. Dermatol..

[61] Müller F.E., Braun M., Syring I., Klümper N., Schmidt D., Perner S., Hauser S., Müller S.C., Ellinger J. (2015). NDUFA4 expression in clear cell renal cell carcinoma is predictive for cancer-specific survival. Am. J. Cancer Res..

[62] Narimatsu T., Matsuura K., Nakada C., Tsukamoto Y., Hijiya N., Kai T., Inoue T., Uchida T., Nomura T., Sato F. (2015). Downregulation of NDUFB6 due to 9p24.1-p13.3 loss is implicated in metastatic clear cell renal cell carcinoma. Cancer Med..

[63] Poulos R.C., Thoms J.A.I., Shah A., Beck D., Pimanda J.E., Wong J.W.H. (2015). Systematic screening of promoter regions pinpoints functional *cis*-regulatory mutations in a cutaneous melanoma genome. Mol. Cancer Res..

[64] Li L.D., Sun H.F., Liu X.X., Gao S.P., Jiang H.L., Hu X., Jin W. (2015). Down-regulation of NDUFB9 promotes breast cancer cell proliferation, metastasis by mediating mitochondrial metabolism. PLoS ONE.

[65] He X., Zhou A., Lu H., Chen Y., Huang G., Yue X., Zhao P., Wu Y. (2013). Suppression of mitochondrial complex I influences cell metastatic properties. PLoS ONE.

[66] Santidrian A.F., Matsuno-Yagi A., Ritland M., Seo B.B., LeBoeuf S.E., Gay L.J., Yagi T., Felding-Habermann B. (2013). Mitochondrial complex I activity and NAD+/NADH balance regulate breast cancer progression. J. Clin. Investig..

[67] Nallar S.C., Kalvakolanu D.V. (2017). GRIM-19: A master regulator of cytokine induced tumor suppression, metastasis and energy metabolism. Cytokine Growth Factor Rev..

[68] Angell J.E., Lindner D.J., Shapiro P.S., Hofmann E.R., Kalvakolanu D.V. (2000). Identification of GRIM-19, a novel cell death-regulatory gene induced by the interferon-β and retinoic acid combination, using a genetic approach. J. Biol. Chem..

[69] Fearnley I.M., Carroll J., Shannon R.J., Runswick M.J., Walker J.E., Hirst J. (2001). GRIM-19, a cell death regulatory gene product, is a subunit of bovine mitochondrial NADH: Ubiquinone oxidoreductase (complex I). J. Biol. Chem..

[70] Huang G., Lu H., Hao A., Ng D.C.H., Ponniah S., Guo K., Lufei C., Zeng Q., Cao X. (2004). GRIM-19, a cell death regulatory protein, is essential for assembly and function of mitochondrial complex I. Mol. Cell. Biol..

[71] Kalakonda S., Nallar S.C., Jaber S., Keay S.K., Rorke E., Munivenkatappa R., Lindner D.J., Fiskum G.M., Kalvakolanu D.V. (2013). Monoallelic loss of tumor suppressor GRIM-19 promotes tumorigenesis in mice. Proc. Natl. Acad. Sci. USA.

[72] Lufei C., Ma J., Huang G., Zhang T., Novotny-Diermayr V., Ong C.T., Cao X. (2003). GRIM-19, a death-regulatory gene product, suppresses Stat3 activity via functional interaction. EMBO J..

[73] Kalakonda S., Nallar S.C., Lindner D.J., Hu J., Reddy S.P., Kalvakolanu D.V. (2007). Tumor-suppressive activity of the cell death activator GRIM-19 on a constitutively active signal transducer and activator of transcription 3. Cancer Res..

[74] Yu H., Lee H., Herrmann A., Buettner R., Jove R. (2014). Revisiting STAT3 signalling in cancer: New and unexpected biological functions. Nat. Rev. Cancer.

[75] Li F., Ren W., Zhao Y., Fu Z., Ji Y., Zhu Y., Qin C. (2012). Downregulation of GRIM-19 is associated with hyperactivation of p-STAT3 in hepatocellular carcinoma. Med. Oncol. Northwood Lond. Engl..

[76] Zhou Y., Li M., Wei Y., Feng D., Peng C., Weng H., Ma Y., Bao L., Nallar S., Kalakonda S. (2009). Down-regulation of GRIM-19 expression is associated with hyperactivation of STAT3-induced gene expression and tumor growth in human cervical cancers. J. Interferon Cytokine Res. Off. J. Int. Soc. Interferon Cytokine Res..

[77] Gong L.B., Luo X.L., Liu S.Y., Tao D.D., Gong J.P., Hu J.B. (2007). Correlations of GRIM-19 and its target gene product STAT3 to malignancy of human colorectal carcinoma. Ai Zheng.

[78] Alchanati I., Nallar S.C., Sun P., Gao L., Hu J., Stein A., Yakirevich E., Konforty D., Alroy I., Zhao X. (2006). A proteomic analysis reveals the loss of expression of the cell death regulatory gene GRIM-19 in human renal cell carcinomas. Oncogene.

[79] Zhou T., Chao L., Rong G., Wang C., Ma R., Wang X. (2013). Down-regulation of GRIM-19 is associated with STAT3 overexpression in breast carcinomas. Hum. Pathol..

[80] Máximo V., Botelho T., Capela J., Soares P., Lima J., Taveira A., Amaro T., Barbosa A.P., Preto A., Harach H.R. (2005). Somatic and germline mutation in GRIM-19, a dual function gene involved in mitochondrial metabolism and cell death, is linked to mitochondrion-rich (Hurthle cell) tumours of the thyroid. Br. J. Cancer.

[81] Suhane S., Berel D., Ramanujan V.K. (2011). Biomarker signatures of mitochondrial NDUFS3 in invasive breast carcinoma. Biochem. Biophys. Res. Commun..

[82] Su C.-Y., Chang Y.-C., Yang C.-J., Huang M.-S., Hsiao M. (2016). The opposite prognostic effect of NDUFS1 and NDUFS8 in lung cancer reflects the oncojanus role of mitochondrial complex I. Sci. Rep..

[83] Porcelli A.M., Ghelli A., Ceccarelli C., Lang M., Cenacchi G., Capristo M., Pennisi L.F., Morra I., Ciccarelli E., Melcarne A. (2010). The genetic and metabolic signature of oncocytic transformation implicates HIF1α destabilization. Hum. Mol. Genet..

[84] Gasparre G., Porcelli A.M., Bonora E., Pennisi L.F., Toller M., Iommarini L., Ghelli A., Moretti M., Betts C.M., Martinelli G.N. (2007). Disruptive mitochondrial DNA mutations in complex I subunits are markers of oncocytic phenotype in thyroid tumors. Proc. Natl. Acad. Sci. USA.

[85] Gasparre G., Hervouet E., de Laplanche E., Demont J., Pennisi L.F., Colombel M., Mège-Lechevallier F., Scoazec J.Y., Bonora E., Smeets R. (2008). Clonal expansion of mutated mitochondrial DNA is associated with tumor formation and complex I deficiency in the benign renal oncocytoma. Hum. Mol. Genet..

[86] Bonora E., Porcelli A.M., Gasparre G., Biondi A., Ghelli A., Carelli V., Baracca A., Tallini G., Martinuzzi A., Lenaz G. (2006). Defective oxidative phosphorylation in thyroid oncocytic carcinoma is associated with pathogenic mitochondrial DNA mutations affecting complexes I and III. Cancer Res..

[87] Palorini R., Simonetto T., Cirulli C., Chiaradonna F. (2013). Mitochondrial complex I inhibitors and forced oxidative phosphorylation synergize in inducing cancer cell death. Int. J. Cell Biol..

[88] Fato R., Bergamini C., Bortolus M., Maniero A.L., Leoni S., Ohnishi T., Lenaz G. (2009). Differential effects of mitochondrial Complex I inhibitors on production of reactive oxygen species. Biochim. Biophys. Acta.

[89] Evans J.M.M., Donnelly L.A., Emslie-Smith A.M., Alessi D.R., Morris A.D. (2005). Metformin and reduced risk of cancer in diabetic patients. BMJ.

[90] Bowker S.L., Majumdar S.R., Veugelers P., Johnson J.A. (2006). Increased cancer-related mortality for patients with type 2 diabetes who use sulfonylureas or insulin. Diabetes Care.

[91] Dowling R.J.O., Niraula S., Stambolic V., Goodwin P.J. (2012). Metformin in cancer: Translational challenges. J. Mol. Endocrinol..

[92] Ellinghaus P., Heisler I., Unterschemmann K., Haerter M., Beck H., Greschat S., Ehrmann A., Summer H., Flamme I., Oehme F. (2013). BAY 87-2243, a highly potent and selective inhibitor of hypoxia-induced gene activation has antitumor activities by inhibition of mitochondrial complex I. Cancer Med..

[93] Bastian A., Matsuzaki S., Humphries K.M., Pharaoh G.A., Doshi A., Zaware N., Gangjee A., Ihnat M.A. (2017). AG311, a small molecule inhibitor of complex I and hypoxia-induced HIF-1α stabilization. Cancer Lett..

[94] Wheaton W.W., Weinberg S.E., Hamanaka R.B., Soberanes S., Sullivan L.B., Anso E., Glasauer A., Dufour E., Mutlu G.M., Budigner G.S. (2014). Metformin inhibits mitochondrial complex I of cancer cells to reduce tumorigenesis. eLife.

[95] Schöckel L., Glasauer A., Basit F., Bitschar K., Truong H., Erdmann G., Algire C., Hägebarth A., Willems P.H., Kopitz C. (2015). Targeting mitochondrial complex I using BAY 87-2243 reduces melanoma tumor growth. Cancer Metab..

[96] Storozhuk Y., Hopmans S.N., Sanli T., Barron C., Tsiani E., Cutz J.-C., Pond G., Wright J., Singh G., Tsakiridis T. (2013). Metformin inhibits growth and enhances radiation response of non-small cell lung cancer (NSCLC) through ATM and AMPK. Br. J. Cancer.

[97] Prior S., Kim A., Yoshihara T., Tobita S., Takeuchi T., Higuchi M. (2014). Mitochondrial respiratory function induces endogenous hypoxia. PLoS ONE.

[98] Brunmair B., Lest A., Staniek K., Gras F., Scharf N., Roden M., Nohl H., Waldhäusl W., Fürnsinn C. (2004). Fenofibrate impairs rat mitochondrial function by inhibition of respiratory complex I. J. Pharmacol. Exp. Ther..

[99] Wilk A., Wyczechowska D., Zapata A., Dean M., Mullinax J., Marrero L., Parsons C., Peruzzi F., Culicchia F., Ochoa A. (2015). Molecular mechanisms of fenofibrate-induced metabolic catastrophe and glioblastoma cell death. Mol. Cell. Biol..

[100] Gui D.Y., Sullivan L.B., Luengo A., Hosios A.M., Bush L.N., Gitego N., Davidson S.M., Freinkman E., Thomas C.J., Vander Heiden M.G. (2016). Environment dictates dependence on mitochondrial complex I for NAD+ and aspartate production and determines cancer cell sensitivity to metformin. Cell Metab..

[101] Weinberg S.E., Chandel N.S. (2015). Targeting mitochondria metabolism for cancer therapy. Nat. Chem. Biol..

[102] Guimarães T.A., Farias L.C., Santos E.S., de Carvalho Fraga C.A., Orsini L.A., de Freitas Teles L., Feltenberger J.D., de Jesus S.F., de Souza M.G., Santos S.H.S. (2016). Metformin increases PDH and suppresses HIF-1α under hypoxic conditions and induces cell death in oral squamous cell carcinoma. Oncotarget.

[103] Liu Z., Ren L., Liu C., Xia T., Zha X., Wang S. (2015). Phenformin induces cell cycle change, apoptosis, and mesenchymal–epithelial transition and regulates the AMPK/mTOR/p70S6K and MAPK/ERK pathways in breast cancer cells. PLoS ONE.

[104] Appleyard M.V.C.L., Murray K.E., Coates P.J., Wullschleger S., Bray S.E., Kernohan N.M., Fleming S., Alessi D.R., Thompson A.M. (2012). Phenformin as prophylaxis and therapy in breast cancer xenografts. Br. J. Cancer.

[105] Narise K., Okuda K., Enomoto Y., Hirayama T., Nagasawa H. (2014). Optimization of biguanide derivatives as selective antitumor agents blocking adaptive stress responses in the tumor microenvironment. Drug Des. Devel. Ther..

[106] Bastian A., Thorpe J.E., Disch B.C., Bailey-Downs L.C., Gangjee A., Devambatla R.K.V., Henthorn J., Humphries K.M., Vadvalkar S.S., Ihnat M.A. (2015). A small molecule with anticancer and antimetastatic activities induces rapid mitochondrial-associated necrosis in breast cancer. J. Pharmacol. Exp. Ther..

[107] Akatsuka A., Kojima N., Okamura M., Dan S., Yamori T. (2016). A novel thiophene-3-carboxamide analog of annonaceous acetogenin exhibits antitumor activity via inhibition of mitochondrial complex I. Pharmacol. Res. Perspect..

[108] Chen G., Zhang X., Zhao M., Wang Y., Cheng X., Wang D., Xu Y., Du Z., Yu X. (2011). Celastrol targets mitochondrial respiratory chain complex I to induce reactive oxygen species-dependent cytotoxicity in tumor cells. BMC Cancer.

[109] Morgan J.B., Liu Y., Coothankandaswamy V., Mahdi F., Jekabsons M.B., Gerwick W.H., Valeriote F.A., Zhou Y.D., Nagle D.G. (2015). Kalkitoxin inhibits angiogenesis, disrupts cellular hypoxic signaling, and blocks mitochondrial electron transport in tumor cells. Mar. Drugs.

[110] Jeso V., Yang C., Cameron M.D., Cleveland J.L., Micalizio G.C. (2013). Synthesis and SAR of Lehualide B: A marine-derived natural product with potent anti-multiple myeloma activity. ACS Chem. Biol..

[111] Tan A.S., Baty J.W., Dong L.F., Bezawork-Geleta A., Endaya B., Goodwin J., Bajzikova M., Kovarova J., Peterka M., Yan B. (2015). Mitochondrial genome acquisition restores respiratory function and tumorigenic potential of cancer cells without mitochondrial DNA. Cell Metab..

[112] Dong L.F., Kovarova J., Bajzikova M., Bezawork-Geleta A., Svec D., Endaya B., Sachaphibulkij K., Coelho A.R., Sebkova N., Ruzickova A. (2017). Horizontal transfer of whole mitochondria restores tumorigenic potential in mitochondrial DNA-deficient cancer cells. eLife.

